# Hypertension and diabetes, but not leptin and adiponectin, mediate the relationship between body fat and chronic kidney disease

**DOI:** 10.1007/s12020-024-03811-6

**Published:** 2024-04-16

**Authors:** Robin Lengton, Friedo W. Dekker, Elisabeth F. C. van Rossum, Johan W. de Fijter, Frits R. Rosendaal, Ko Willems van Dijk, Ton J. Rabelink, Saskia Le Cessie, Renée de Mutsert, Ellen K. Hoogeveen

**Affiliations:** 1 Department of Clinical Epidemiology, Leiden University Medical Center, Leiden, The Netherlands; 2 Department of Internal Medicine, Division of Endocrinology, Erasmus University Medical Center Rotterdam, Rotterdam, The Netherlands; 3 Department of Nephrology, Leiden University Medical Center, Leiden, The Netherlands; 4 Department of Human Genetics and Medicine, Division of Endocrinology, Leiden University Medical Center, Leiden, The Netherlands; 5 Department of Biomedical Data Sciences, Leiden University Medical Center, Leiden, The Netherlands; 6 Department of Nephrology, Jeroen Bosch Hospital, Den Bosch, The Netherlands

**Keywords:** Obesity, Kidney, Hypertension, Diabetes, Leptin, Adiponectin

## Abstract

**Purpose:**

Obesity may promote kidney damage through hemodynamic and hormonal effects. We investigated the association between body mass index (BMI), total body fat (TBF) and chronic kidney disease (CKD) and whether hypertension, diabetes, leptin and adiponectin mediated these associations.

**Methods:**

In this cross-sectional analysis of the Netherlands Epidemiology of Obesity study, 6671 participants (45–65 y) were included. We defined CKD as eGFR <60 ml/min/1.73 m^2^ and/or moderately increased albuminuria. The percentage of mediation was calculated using general structural equation modeling, adjusted for potential confounding factors age, sex, smoking, ethnicity, physical activity and Dutch healthy diet index.

**Results:**

At baseline mean (SD) age was 56 (6), BMI 26.3 (4.4), 44% men, and 4% had CKD. Higher BMI and TBF were associated with 1.08 (95%CI 1.05; 1.11) and 1.05-fold (95%CI 1.02; 1.08) increased odds of CKD, respectively. As adiponectin was not associated with any of the outcomes, it was not studied further as a mediating factor. The association between BMI and CKD was 8.5% (95%CI 0.5; 16.5) mediated by diabetes and 22.3% (95%CI 7.5; 37.2) by hypertension. In addition, the association between TBF and CKD was 9.6% (95%CI −0.4; 19.6) mediated by diabetes and 22.4% (95%CI 4.2; 40.6) by hypertension. We could not confirm mediation by leptin in the association between BMI and CKD (35.6% [95%CI −18.8; 90.3]), nor between TBF and CKD (59.7% [95%CI −7.1; 126.6]).

**Conclusion:**

Our results suggest that the relations between BMI, TBF and CKD are in part mediated by diabetes and hypertension.

## Introduction

Globally, the prevalence of obesity (body mass index (BMI) ≥ 30 kg/m^2^) has tripled since 1975 [1, 2]. Approximately 604 million adults suffered from obesity worldwide in 2015 [3]. Obesity is related with an increased risk of hypertension and type 2 diabetes mellitus (T2D), the leading causes of chronic kidney disease (CKD) [4]. CKD is a major public health burden with a global prevalence of about 11% in adults [5, 6]. CKD is an important risk factor of kidney failure, cardiovascular disease, lower quality of life and mortality [7, 8].

Obesity may promote kidney damage through both hemodynamic and hormonal effects, contributing to glomerular and interstitial fibrosis [9]. It is hypothesized that the deleterious effects of obesity on the kidney are, in part, mediated by cardiovascular risk factors such as T2D, dyslipidemia and hypertension [10]. Moreover, obesity is associated with an increased single-nephron glomerular filtration rate, which may lead to glomerulosclerosis and subsequent albuminuria and loss of kidney function over time [11, 12]. In individuals with obesity it is observed that measures of body fat distribution are strongly associated with cardiometabolic risk factors [13, 14]. Rapid expansion of adipose tissue can increase production of pro-inflammatory cytokines and down-regulate the production of anti-inflammatory hormones, such as leptin and adiponectin [9]. Synthesis of adipokines by body fat can stimulate the sympathetic nerve activity and thereby activate the renin-angiotensin-aldosterone system (RAAS), which is an important risk factor of hypertension [15]. In addition to subcutaneous fat, especially visceral fat may be an important risk factor for albuminuria [12]. The mechanisms linking total body fat (TBF) distribution and CKD need to be addressed.

**Fig. 1:**
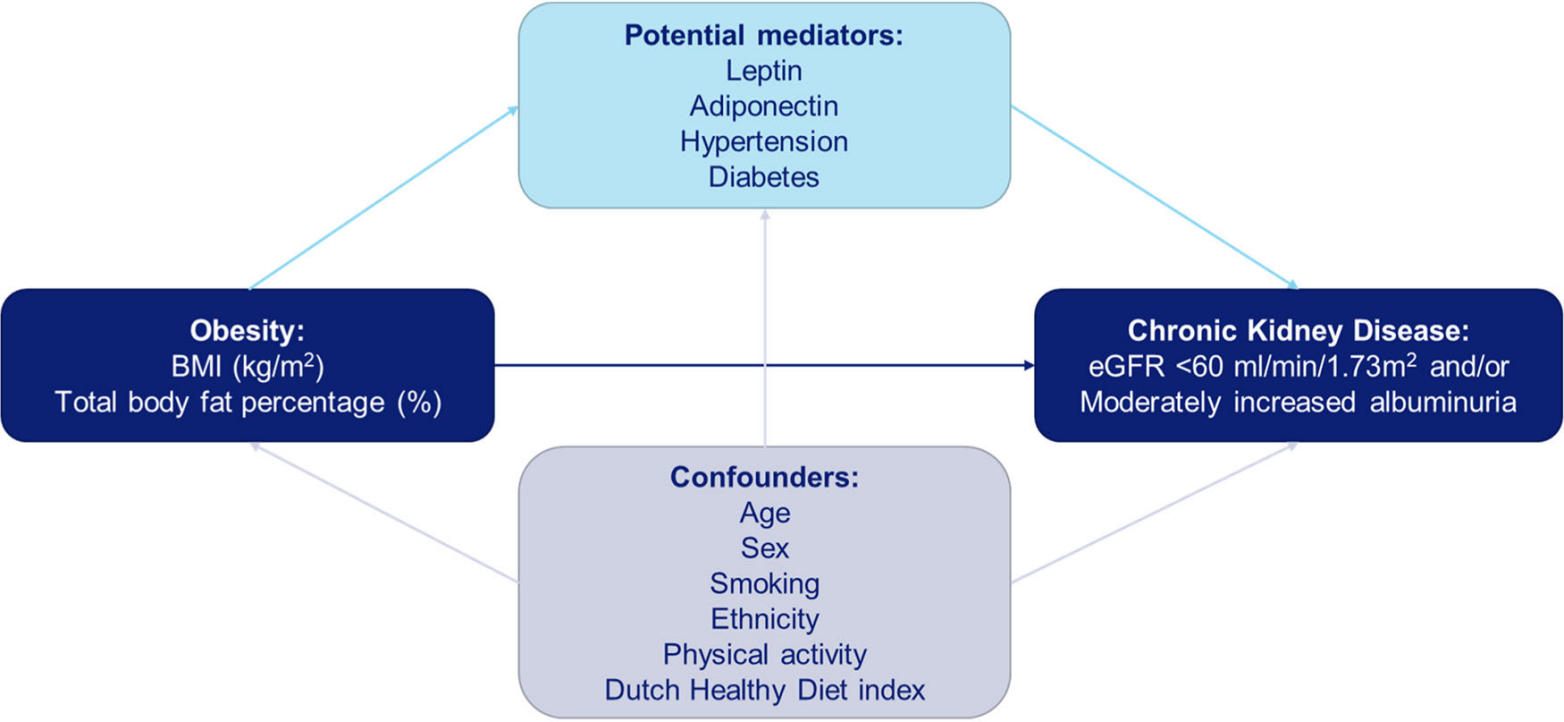
Hypothesis path diagram of the studied association between body fat and chronic kidney disease and the potential mediators leptin, adiponectin, hypertension and diabetes. BMI body mass index, eGFR estimated glomerular filtration rate

It remains unclear to what extent both hemodynamic (hypertension and diabetes) and hormonal (leptin and adiponectin) effects mediate the association between body fat and CKD. We used both high BMI and TBF as a proxy for obesity to investigate the association with CKD defined as eGFR < 60 ml/min/1.73 m^2^ and/or moderately increased albuminuria. We specifically examined to what extent leptin and adiponectin, as well as hypertension and diabetes, play a mediating role in these associations (Fig. 1).

## Materials and methods

### Study design and population

The present study is a cross-sectional analysis of baseline measurements of the Netherlands Epidemiology of Obesity (NEO) study, a population-based, prospective cohort study of individuals (45–65 y) included between 2008 and 2012, with an oversampling of individuals with overweight or obesity, living in the greater area of Leiden (in the West of the Netherlands). Inhabitants (45–65 y) from the adjacent Leiderdorp municipality were included irrespective of their BMI, allowing for a reference distribution of BMI. In total, 6671 participants were included in the study. The study design and population have been described in detail elsewhere [16]. The Medical Ethical Committee of the Leiden University Medical Center (LUMC) approved the design of the study (protocol number:P08.109). All participants gave written informed consent.

### Data collection

Participants were invited to a baseline visit after an overnight fast. Prior to this visit, participants completed questionnaires regarding information on demographic, lifestyle, clinical, diet and physical activity. Participants reported habitual dietary intake using a semi-quantitative self-administered 125-item food-frequency questionnaire [17, 18]. Subsequently, the Dutch healthy diet index was calculated, which indicated adherence to the Dutch Guidelines for a Healthy Diet of 2015 [19]. The duration and frequency of physical activity during leisure time was reported by participants in the Short QUestionnaire to ASsess Health-enhancing physical activity (SQUASH) [20, 21]. At the baseline visit an extensive physical examination was performed, medication was registered and blood samples were drawn. Diabetes was defined as self-reported physician’s diagnosis, use of glucose lowering drugs or fasting glucose levels ≥7 mmol/L. Hypertension was defined as systolic blood pressure ≥140 mmHg, diastolic blood pressure ≥90 mmHg or use of antihypertensive drugs. Cardiovascular disease was defined as history of myocardial infarction, angina pectoris or congestive heart failure. Ethnicity and smoking status were self-reported. Highest level of education was categorized as: low education (no education/primary school/lower-vocational-education) or high education (higher-vocational-education/university/postgraduate-education).

### Body weight and total body fat

At the baseline visit height was measured, without shoes, using a vertically fixed calibrated tape measure. Subsequently, without shoes, body weight was measured and TBF was estimated by the Tanita bio impedance balance (TBF-310, Tanita International Division, UK). To correct for the weight of clothing, 1 kg was subtracted. BMI at baseline was calculated by dividing the weight in kg by the height in meters squared. Participants were divided into three BMI categories according to WHO criteria: normal weight (BMI < 25 kg/m^2^), overweight (BMI ≥ 25 & < 30 kg/m^2^) and obesity (BMI ≥ 30 kg/m^2^) [1].

### Assessment of leptin and adiponectin concentrations

Serum leptin concentrations were measured using a human leptin competitive RadioImmunoAssay (RIA) (CatNr:HL-81HK, Merck Millipore, Darmstadt, Germany). The concentrations were counted using a gamma counter (Wizard 2 3470, Perkin Elmer, StatLia software). Coefficients of leptin variation were calculated based on 22 runs over 105 days and were 12–14% at concentrations between 19 and 55 μg/L. The concentrations of adiponectin were measured in serum using a latex particle-enhanced turbidimetric immunoassay (CatNr:A0299, Randox Laboratories Limited) on an automated analyzer (Roche Modular P800) [22].

### Kidney function and CKD

At baseline, serum creatinine concentrations were measured from fasting blood samples using Jaffe kinetic compensated method between September 1st 2008 and November 30th 2010 and an enzymatic assay (IDMS calibrated against SRM 967) from December 1st 2010 until the end of the inclusion period [16]. Serum Jaffe results were corrected with a fixed compensation factor of −26 µmol/L to compensate for assay non-specificity. From these levels creatinine-based estimated glomerular filtration rate (eGFR) was calculated using the 2012 CKD Epidemiology Collaboration (CKD-EPI) equation, taking into account age, sex, and race [23]. Albuminuria was measured using spot morning urine samples, whereby albumin was measured using an immunoturbidimetric assay and creatinine using the same methods as for serum creatinine. Because urinary creatinine concentrations are not affected by pseudochromogens they are exchangeable using either a Jaffe or an enzymatic method. Moderately increased albuminuria was defined as urinary albumin-creatinine ratio (UACR) of ≥2.5 mg/mmol in men and of ≥3.5 mg/mmol in women [24]. We defined CKD as an eGFR < 60 mL/min/1.73 m^2^ and/or moderately increased albuminuria [25].

### Statistical analysis

Individuals with a BMI ≥ 27 kg/m^2^ were oversampled in the NEO study. To correctly represent distributions and associations in the general population [26], adjustments for the oversampling were made by weighting the analyses towards the BMI distribution of participants from the Leiderdorp municipality [27], whose BMI distribution was similar to the general Dutch population [28]. All results were based on weighted analyses. Consequently, results apply to a population-based study without oversampling of individuals with BMI ≥ 27 kg/m^2^. Baseline characteristics are presented for all 6671 participants and stratified by BMI. Data are presented as mean with standard deviation (SD), median (interquartile range) or percentages, depending on the underlying distribution. As a consequence of the weighted analyses, no absolute numbers could be given, only proportions. Analyses were performed based on complete cases.

Since BMI is subject to misclassification of body fat, due to its relation to height and muscle mass, we aimed to investigate the association of both BMI and TBF with CKD. We studied by logistic regression the relation between exposure (BMI or TBF) and outcome (CKD or eGFR < 60 ml/min/1.73m^2^ or moderately increased albuminuria), adjusted for important confounding factors age, sex, smoking, ethnicity, physical activity and Dutch healthy diet index. Subsequently, to investigate whether leptin, adiponectin, hypertension and diabetes mediate the effect of exposure on outcome (Fig. 1), the method proposed by Baron and Kenny [29] was used. First, we checked whether associations between exposure-outcome, exposure-mediator and mediator-outcome were present. When investigating the associations between BMI or TBF with leptin and adiponectin, residuals were not normally distributed. To be able to perform linear regression analysis, adiponectin and leptin concentrations underwent natural logarithmic transformation, using the mathematical constant “*e”* which is about 2.7. The logarithm of leptin and adiponectin was used for all further analyses. The results in the linear regression analyses were back-transformed and can be interpreted as the relative change per 1 kg/m^2^ BMI or per 1% TBF. The logistic regression analyses can be interpreted as the change in odds of BMI and TBF per 2.7-fold increase in the concentrations of leptin and adiponectin. Second, we checked if there was no interaction between the exposure and mediators. Confounding between mediator and outcome was attempted to be handled by adjusting for potential confounding factors: age, sex, smoking (current, former, or never), ethnicity, physical activity and Dutch healthy diet index (full model). If all associations were present and there was no interaction, we compared the regression coefficient of the association between exposure-outcome (estimated total effect) with the regression coefficient after controlling for the different mediators (leptin, adiponectin, hypertension and diabetes) (estimated direct effect).

Finally, we used general structural equation modeling (GSEM) to calculate the percentage of mediation with the corresponding 95% confidence intervals (CIs) of the different mediators in the relation between BMI or TBF and CKD, adjusted for the potential confounders [30]. The 95% CIs convey the essential information by indicating the range of values that are reasonably compatible with the observations. The 95% CIs convey information about the size and precision of the point estimate (or effect), determined by the size of the study sample [31, 32]. By multiplying the regression coefficients of the model’s exposure-mediator and mediator-outcome associations, the separate and combined indirect effects of the different mediators on the association between obesity and CKD could be calculated. Subsequently, we divided the indirect effects by the total effect, calculated as the sum of the direct and indirect effects, to calculate the percentage of mediation by the different mediators.

All statistical analysis were performed using STATA/SE, version 16.

## Results

### Baseline characteristics

Baseline characteristics of all participants (*N* = 6671) of the NEO study, and according to three BMI categories, are presented in Table 1. According to the WHO criteria, 43% of the participants were overweight (BMI ≥ 25 and <30 kg/m^2^) and 45% had obesity (BMI ≥ 30 kg/m^2^) [1]. Serum leptin and adiponectin levels were measured in 6609 participants. Mean eGFR of all participants was 86.2 ± 12.4 mL/min/1.73 m^2^ and 2% had an eGFR < 60 mL/min/1.73 m^2^. The prevalence of moderately increased albuminuria was 2% and of CKD 4%. Participants with obesity had lower levels of education, more comorbid conditions and used more medication compared to participants with normal weight or overweight.

**Table 1:** Characteristics of 6671 Middle-Aged participants of the Netherlands Epidemiology of Obesity (NEO) study, stratified by body mass index

Baseline characteristics	All (*N* = 6671)	Normal weight BMI < 25 kg/m^2^ (12%)	Overweight BMI ≥ 25 & < 30 kg/m^2^ (43%)	Obesity BMI ≥ 30 kg/m^2^ (45%)
Demographic/anthropometric				
Age, y	56 ± 6	56 ± 3	56 ± 6	56 ± 10
Sex, men (%)	44	34	54	43
Education level, high (%)	46	55	43	30
Smoking, current (%)	16	15	17	16
Alcohol intake, g/d	9.8 (2.7–21.3)	9.4 (3.2–20.9)	10.9 (3.2–22.6)	7.5 (1.0–20.9)
Ethnicity, white (%)	95	95	95	94
Body mass index, kg/m^2^	26.3 ± 4.4	22.6 ± 0.8	27.1 ± 1.4	34.0 ± 6.6
Body weight, kg	79.2 ± 15.9	67.6 ± 5	82.7 ± 10.3	100.7 ± 25.3
Waist circumference, cm				
Men	98.4 ± 11.4	88.7 ± 2.9	99.6 ± 6.6	115.0 ± 15.4
Women	87.4 ± 12.7	78.2 ± 3.6	91.2 ± 7.4	107.1 ± 18.1
Total body fat, %				
Men	25.0 ± 6.1	19.6 ± 1.6	25.8 ± 3.3	33.8 ± 9.0
Women	36.9 ± 6.4	32.1 ± 2.5	39.5 ± 3.3	46.1 ± 6.1
Blood pressure, mm Hg				
Systolic blood pressure	130.1 ± 17.0	127.3 ± 8.9	131.4 ± 16.9	134.1 ± 28.7
Diastolic blood pressure	83.1 ± 10.3	80.8 ± 5.2	84.2 ± 10.3	86.3 ± 17.2
Comorbidity				
Diabetes^a^ (%)	6	2	7	17
Hypertension^b^ (%)	46	35	50	66
Cardiovascular disease^c^ (%)	6	4	6	8
Chronic kidney disease^d^ (%)	4	3	5	7
Medication use				
Glucose-lowering drugs^e^ (%)	3	1	2	9
Anti-hypertensive drugs^f^ (%)	24	16	25	41
Corticosteroid use^g^ (%)	9	8	9	12
Laboratory Measurements				
Serum creatinine, µmol/L	75 (66–85)	73 (65–82)	78 (69–88)	74 (66–85)
eGFR^h^, mL/min/1.73 m^2^	86.2 ± 12.4	86.5 ± 6.4	85.8 ± 12.7	86.7 ± 22.0
eGFR^h^, <60 mL/min/1.73 m^2^ (%)	2	2	2	3
Leptin, ug/L	12.3 (6.7–22.7)	8.8 (4.9–14.4)	13 (7.3–24.3)	30.8 (17–46.8)
Adiponectin, mg/L	8.3 (5.7–11.9)	10.0 (6.9–13.4)	7.4 (5.2–10.6)	7.0 (4.8–10)
Total cholesterol, mmol/L	5.6 (5–6.3)	5.6 (5–6.3)	5.7 (5–6.4)	5.6 (4.8–6.3)
HDL cholesterol, mmol/L	1.5 (1.2–1.9)	1.8 (1.4–2.1)	1.4 (1.2–1.7)	1.3 (1.1–1.6)
LDL cholesterol, mmol/L	3.5 (2.9–4.1)	3.4 (2.8–4)	3.7 (3–4.3)	3.5 (2.8–4.1)
Fasting glucose, mmol/L	5.3 (5–5.7)	5.1 (4.8–5.4)	5.4 (5.1–5.8)	5.7 (5.3–6.2)
Fasting insulin, mU/l	7.8 (5.2–11.8)	5.9 (4.2–8.2)	8.8 (6.2–12.3)	13.7 (9.3–20.1)
Triglycerides, mmol/L	1 (0.7–1.5)	0.8 (0.6–1.2)	1.1 (0.8–1.6)	1.4 (1–1.9)
Urine				
UACR, mg/mmol	0.4 (0.3–0.7)	0.5 (0.3–0.7)	0.4 (0.3–0.7)	0.5 (0.3–0.8)
Moderatey increased albuminuria^i^ (%)	2	1	3	5

Data are presented as mean (SD), median (25th–75th percentile/range) or percentage. Results were based on analyses weighted towards the body mass index distribution of the general population

*eGFR* estimated glomerular filtration rate, *HDL* high density lipoprotein, *LDL* low density lipoprotein, *UACR* urine albumin-to-creatinine ratio

^a^Diabetes mellitus is considered present in case of a self-reported physician’s diagnosis and/or use of glucose lowering drugs or fasting glucose levels ≥ 7 mmol/L

^b^Systolic blood pressure ≥ 140 mmHg and/or the diastolic blood pressure ≥ 90 mmHg or use of antihypertensive drugs

^c^Cardiovascular disease is considered to be present in case of a history of myocardial infarction, angina pectoris or congestive heart failure

^d^Defined as having an eGFR < 60 mL/min/1.73 m^2^ and/or moderately increased albuminuria

^e^Glucose lowering drugs: ATC codes A10A and A10B

^f^Anti-hypertensive drugs: ATC codes C02, C02A, C02CA, C02D, C03, C07, C08, C09, C09A, C09D

^g^Including oral, inhalation and systemic corticosteroids, as well as fixed dose combinations containing corticosteroids

^h^Creatinine-based eGFR calculated using the 2012 CKD Epidemiology Collaboration (CKD-EPI) equation, taking into account age, sex, and race

^i^Moderately increased albuminuria is defined as UACR ≥ 2.5 mg/mmol in men and ≥ 3.5 mg/mmol in women

### BMI, total body fat and CKD

As shown in Table 2, both BMI and TBF were positively associated with CKD. After multivariable adjustment, per 1 kg/m^2^ BMI (OR 1.08, 95%CI: 1.05; 1.11) or per 1% TBF (OR 1.05, 95%CI: 1.02; 1.08) was associated with an increased risk of CKD. Similarly, after multivariable adjustment, per 1 kg/m^2^ BMI (OR 1.11, 95%CI: 1.08; 1.15) or per 1% TBF (OR 1.08, 95%CI: 1.05; 1.12) was associated with an increased risk of moderately increased albuminuria. However, only weak positive associations were observed between BMI, TBF and eGFR < 60 ml/min/1.73 m^2^.

**Table 2:** Logistic regression analysis of the associations of body mass index and total body fat with chronic kidney disease in participants of the Netherlands Epidemiology of Obesity (NEO) study

		Crude		Model 1^a^		Full model^b^	
	*N*	OR	95% CI	OR	95% CI	OR	95% CI
Chronic kidney disease^c^							
BMI, per 1 kg/m^2^	6671	1.08	1.05 to 1.10	1.08	1.05 to 1.11	1.08	1.05 to 1.11
Total body fat, per 1%	6640	1.03	1.01 to 1.05	1.06	1.03 to 1.08	1.05	1.02 to 1.08
eGFR^d^ < 60 ml/min/1.73m^2^							
BMI, per 1 kg/m^2^	6621	1.03	0.99 to 1.07	1.03	0.99 to 1.08	1.02	0.98 to 1.07
Total body fat, per 1%	6590	1.02	0.99 to 1.05	1.02	0.98 to 1.06	1.01	0.97 to 1.05
Moderately increased albuminuria^e^							
BMI, per 1 kg/m^2^	6644	1.11	1.08 to 1.14	1.11	1.08 to 1.15	1.11	1.08 to 1.15
Total body fat, per 1%	6613	1.03	1.01 to 1.06	1.08	1.05 to 1.12	1.08	1.05 to 1.12

Results were based on analyses weighted towards the body mass index distribution of the general population.

*CI* confidence interval, *eGFR* estimated glomerular filtration rate, *BMI* body mass index

^a^Adjusted for age, sex, smoking and ethnicity.

^b^Model 1 plus additional adjustment for physical activity and Dutch Healthy Diet index

^c^Defined as having an eGFR <60 mL/min/1.73 m^2^ and/or moderately increased albuminuria

^d^Creatinine-based eGFR calculated using the 2012 CKD Epidemiology Collaboration (CKD-EPI) equation, taking into account age, sex, and race

^e^Moderately increased albuminuria is defined as urine albumin-to-creatinine ratio ≥ 2.5 mg/mmol in men and ≥ 3.5 mg/mmol in women

Furthermore, BMI was positively associated with diabetes and hypertension. After multivariable adjustment, per 1 kg/m^2^ BMI was associated with a 1.17-fold increased risk of diabetes (95%CI: 1.15; 1.19) and a 1.11-fold increased risk of hypertension (95%CI: 1.10; 1.13) (Table 3a). In addition, positive associations were observed per 1% TBF with diabetes (OR 1.14, 95%CI: 1.12; 1.17) and hypertension (OR 1.07, 95%CI: 1.05; 1.08) (Table 3a). Using linear regression analyses, BMI and TBF also showed associations with leptin and adiponectin (Table 3b). Per 1 kg/m^2^ BMI was associated with 1.121-fold increased leptin concentrations (95%CI: 1.116; 1.126) and 0.975-fold decreased adiponectin concentrations (95%CI: 0.972; 0.978). Moreover, per 1% TBF was associated with 1.090-fold increased leptin concentrations (95%CI: 1.086; 1.094) and 0.982-fold decreased adiponectin concentrations (95%CI: 0.980; 0.984).

**Table 3:** (a) Logistic regression analysis of the associations of body mass index and total body fat with the binary mediators diabetes or hypertension in participants of the Netherlands Epidemiology of Obesity (NEO) study. (b) Linear regression analysis of the association of body mass index and total body fat with the continuous mediators leptin and adiponectin in participants of the Netherlands Epidemiology of Obesity (NEO) study

(a)							
		Crude		Model 1^a^		Full model^b^	
	N	OR	95% CI	OR	95% CI	OR	95% CI
Diabetes^**c**^							
BMI, per 1 kg/m^2^	6671	1.16	1.14 to 1.18	1.18	1.15 to 1.20	1.17	1.15 to 1.19
Total body fat, per 1%	6640	1.06	1.04 to 1.07	1.15	1.12 to 1.17	1.14	1.12 to 1.17
Hypertension^**d**^							
BMI, per 1 kg/m^2^	6671	1.11	1.10 to 1.13	1.12	1.10 to 1.13	1.11	1.10 to 1.13
Total body fat, per 1%	6640	1.02	1.01 to 1.03	1.07	1.06 to 1.09	1.07	1.05 to 1.08

(b)							
		Crude		Model 1^a^		Full model^b^	
	*N*	Relative change	95% CI	Relative change	95% CI	Relative change	95% CI
Leptin (%)							
BMI, per 1 kg/m^2^	6609	1.110	1.104 to 1.116	1.124	1.119 to 1.129	1.121	1.116 to 1.126
Total body fat, per 1%	6578	1.088	1.085 to 1.091	1.092	1.088 to 1.096	1.090	1.086 to 1.094
Adiponectin (%)							
BMI, per 1 kg/m^2^	6609	0.968	0.965 to 0.972	0.974	0.972 to 0.977	0.975	0.972 to 0.978
Total body fat, per 1%	6578	1.012	1.010 to 1.014	0.981	0.979 to 0.984	0.982	0.980 to 0.984

Results were based on analyses weighted towards the body mass index distribution of the general population. Leptin and adiponectin were logarithmic transformed.

*CI* confidence interval, *BMI* body mass index

^a^Adjusted for age, sex, smoking and ethnicity

^b^Model 1 plus additional adjustment for physical activity and Dutch Healthy Diet index

^c^Diabetes mellitus is considered present in case of a self-reported physician’s diagnosis and/or use of glucose lowering drugs or fasting glucose levels ≥7 mmol/L

^d^Systolic blood pressure ≥140 mmHg and/or the diastolic blood pressure ≥90 mmHg or use of antihypertensive drugs

Table 4 shows the associations between the different mediators (leptin, adiponectin, diabetes and hypertension) and CKD, and separately eGFR < 60 ml/min/1.73 m^2^ or moderately increased albuminuria. No associations were observed between adiponectin and any of the outcomes. After multivariable adjustment, each 2.7-fold increase in leptin (μg/L) was associated with an increased risk of CKD (OR 1.62, 95%CI: 1.23; 2.14), eGFR < 60 ml/min/1.73m^2^ (OR 1.74, 95%CI: 1.18; 1.57) and moderately increased albuminuria (OR 1.57, 95%CI: 1.09; 2.28). Subsequently, having diabetes was associated with a 2.2-fold increased odds of CKD (95%CI: 1.44; 3.23) and 2.6-fold increased odds of moderately increased albuminuria (95%CI: 1.74; 3.87). The same trend was observed for the risk of eGFR < 60 ml/min/1.73m^2^ (OR 1.80, 95%CI: 0.88; 3.69). In addition, having hypertension was associated with a 2.3-fold increased odds of CKD (95%CI: 1.56; 3.36), 1.8-fold increased odds of eGFR < 60 ml/min/1.73m^2^ (95%CI: 1.06; 3.15) and a 3.0-fold increased odds of moderately increased albuminuria (95%CI: 1.79; 5.06).

**Table 4:** Logistic regression analysis of the association of leptin, adiponectin, diabetes and hypertension with chronic kidney disease in participants of the Netherlands Epidemiology of Obesity (NEO)

		Crude		Model 1^a^		Full model^b^	
	*N*	OR	95% CI	OR	95% CI	OR	95% CI
Chronic kidney disease^c^							
Leptin^d^ (μg/L)	6609	1.40	1.13 to 1.74	1.69	1.28 to 2.22	1.62	1.23 to 2.14
Adiponectin^d^ (mg/L)	6609	0.89	0.66 to 1.21	0.78	0.53 to 1.15	0.80	0.54 to 1.19
Diabetes^e^, yes	6671	2.93	2.01 to 4.28	2.29	1.54 to 3.40	2.16	1.44 to 3.23
Hypertension^f^, yes	6671	2.85	2.00 to 4.08	2.34	1.60 to 3.42	2.29	1.56 to 3.36
eGFR^g^ < 60 ml/min/1.73m^2^							
Leptin^d^ (μg/L)	6584	1.60	1.15 to 2.23	1.86	1.28 to 2.72	1.74	1.18 to 2.57
Adiponectin^d^ (mg/L)	6584	1.15	0.68 to 1.93	0.82	0.42 to 1.59	0.86	0.44 to 1.68
Diabetes^e^, yes	6621	2.38	1.16 to 4.88	1.86	0.91 to 3.81	1.80	0.88 to 3.69
Hypertension^f^, yes	6621	2.54	1.52 to 4.25	1.90	1.10 to 3.25	1.83	1.06 to 3.15
Moderately increased albuminuria^h^							
Leptin^d^ (μg/L)	6583	1.24	0.95 to 1.61	1.59	1.10 to 2.31	1.57	1.09 to 2.28
Adiponectin^d^ (mg/L)	6583	0.72	0.53 to 0.99	0.81	0.55 to 1.21	0.83	0.55 to 1.24
Diabetes^e^, yes	6644	3.42	2.38 to 4.92	2.76	1.85 to 4.10	2.59	1.74 to 3.87
Hypertension^f^, yes	6644	3.33	2.06 to 5.37	3.06	1.82 to 5.13	3.01	1.79 to 5.06

Results were based on analyses weighted towards the body mass index distribution of the general population.

*CI* confidence interval, *eGFR* estimated glomerular filtration rate

^a^Adjusted for age, sex, smoking and ethnicity

^b^Model 1 plus additional adjustment for physical activity and Dutch Healthy Diet index.

^c^Defined as having an eGFR < 60 mL/min/1.73 m^2^ and/or moderately increased albuminuria

^d^Underwent natural logarithmic transformation

^e^Diabetes mellitus is considered present in case of a self-reported physician’s diagnosis and/or use of glucose lowering drugs or fasting glucose levels ≥ 7 mmol/L

^f^Systolic blood pressure ≥ 140 mmHg and/or the diastolic blood pressure ≥ 90 mmHg or use of antihypertensive drugs

^g^Creatinine-based eGFR calculated using the 2012 CKD Epidemiology Collaboration (CKD-EPI) equation, taking into account age, sex, and race

^h^Moderately increased albuminuria is defined as urine albumin-to-creatinine ratio ≥2.5 mg/mmol in men and ≥ 3.5 mg/mmol in women

### Mediation analyses

As BMI and TBF were only weakly associated with eGFR < 60 ml/min/1.73m^2^, we did not continue the mediation analysis for this outcome. Therefore, only mediation analyses have been performed with CKD and moderately increased albuminuria as outcome variables. In addition, adiponectin was not associated with any of the outcomes, and was not studied further as a mediating factor. We did not observe interaction between the mediators (leptin, diabetes and hypertension) and TBF or BMI in the association with CKD or moderately increased albuminuria (Supplementary Table 1).

As shown in Table 5a, additional adjustment for diabetes slightly attenuated the association of BMI (OR 1.07, 95%CI: 1.04; 1.10) or TBF (OR 1.04, 95%CI: 1.01; 1.07) with CKD. A similar attenuation in the effect was shown after adjustment for hypertension (OR 1.06 [95%CI: 1.03; 1.09] and OR 1.04 [95%CI: 1.01; 1.07], respectively). When controlling for both diabetes and hypertension, the affect attenuated to OR 1.05 (95%CI: 1.02; 1.09) for BMI and to OR 1.03 (95%CI: 1.00; 1.06) for TBF in the association with CKD. Furthermore, additional adjustment for leptin in the association of BMI or TBF with CKD, showed attenuation of the effect (OR 1.05 [95%CI: 1.01; 1.10] and OR 1.02 [95%CI: 0.98; 1.06], respectively). In the association of BMI or TBF with moderately increased albuminuria, additional adjustment for diabetes and/or hypertension showed similar results (Table 5b). However, additional adjustment for leptin slightly strengthened the association between BMI or TBF and moderately increased albuminuria (OR 1.13 [95%CI: 1.09; 1.18] and OR 1.10 [95%CI: 1.05; 1.14], respectively), therefore we could not confirm mediation in this specific association.

**Table 5:** (a) Logistic regression analysis of the associations of body mass index and total body fat with chronic kidney disease, after addition of mediators, in participants of the Netherlands Epidemiology of Obesity (NEO). (b) Logistic regression analysis of the associations of body mass index and total body fat with moderately increased albuminuria, after addition of mediators, in participants of the Netherlands Epidemiology of Obesity (NEO)

(a)						
Chronic kidney disease^a^		Starting model^c^	+ Leptin^d^ (μg/L)	+ Diabetes^e^	+ Hypertension^f^	+Diabetes, Hypertension
	*N*	OR (95% CI)	OR (95% CI)	OR (95% CI)	OR (95% CI)	OR (95% CI)
BMI, per 1 kg/m^2^	6531	1.08 (1.05 to 1.11)	1.05 (1.01 to 1.10)	1.07 (1.04 to 1.10)	1.06 (1.03 to 1.09)	1.05 (1.02 to 1.09)
Total body fat, per 1%	6500	1.05 (1.02 to 1.08)	1.02 (0.98 to 1.06)	1.04 (1.01 to 1.07)	1.04 (1.01 to 1.07)	1.03 (1.00 to 1.06)

(b)						
Moderately increased albuminuria^b^		Starting model^c^	+ Leptin^d^ (μg/L)	+ Diabetes^e^	+ Hypertension^f^	+Diabetes, Hypertension
	*N*	OR (95% CI)	OR (95% CI)	OR (95% CI)	OR (95% CI)	OR (95% CI)
BMI, per 1 kg/m^2^	6508	1.11 (1.08 to 1.15)	1.13 (1.09 to 1.18)	1.10 (1.07 to 1.14)	1.09 (1.06 to 1.13)	1.09 (1.05 to 1.12)
Total body fat, per 1%	6477	1.08 (1.05 to 1.12)	1.10 (1.05 to 1.14)	1.07 (1.04 to 1.11)	1.07 (1.03 to 1.10)	1.06 (1.02 to 1.10)

Results were based on analyses weighted towards the body mass index distribution of the general population. There were no interactions between exposures and mediators (*P* > 0.05)

*CI* confidence interval, *BMI* body mass index

^a^Chronic kidney disease is defined as having an eGFR < 60 ml/min/1.73m^2^ and/or moderately increased albuminuria

^b^Moderately increased albuminuria is defined as urine albumin-to-creatinine ratio ≥ 2.5 mg/mmol in men and ≥ 3.5 mg/mmol in women

^c^Starting model (full model) was adjusted for sex, age, smoking, ethnicity, physical activity and Dutch Healthy Diet index

^d^Underwent natural logarithmic transformation

^e^Diabetes mellitus is considered present in case of a self-reported physician’s diagnosis and/or use of glucose lowering drugs or fasting glucose levels ≥ 7 mmol/L

^f^Systolic blood pressure ≥ 140 mmHg and/or the diastolic blood pressure ≥ 90 mmHg or use of antihypertensive drugs

Subsequently, we performed GSEM analyses to estimate the indirect effects of BMI and TBF through leptin, diabetes and hypertension as a percentage of their total effect (Tables 6a/b and 7a/b). When both diabetes and hypertension were included in the models, the percentage of mediation of the total association between BMI and CKD was 6.5% (95%CI: −0.8; 13.9) for diabetes and 22.0% (95%CI: 6.8; 37.3) for hypertension (Table 6a). Similarly, the percentage of mediation of the total association between TBF and CKD was 7.7% (95%CI: −1.3; 16.7) for diabetes and 22.5% (95%CI: 3.6; 41.4) for hypertension (Table 6b). Moreover, when including leptin in the models, the percentage of mediation in the association between BMI and CKD was 35.6% (95%CI: −18.8; 90.3) (Table 6a) and in the association between TBF and CKD 59.7% (95%C: -7.1; 126.6) (Table 6b).

**Table 6:** (a) Analysis of indirect effects at baseline of the mediators leptin, diabetes and hypertension in the association between body mass index and chronic kidney disease of 6531 participants of the Netherlands Epidemiology of Obesity (NEO). (b) Analysis of indirect effects of the mediators leptin, diabetes and hypertension in the association between total body fat and chronic kidney disease of 6500 participants of the Netherlands Epidemiology of Obesity (NEO)

(a)			
		% of Total Effect	95% CI
Indirect effect^a^ through:			
Leptin^b^ alone		35.6	−18.8 to 90.3
Diabetes^c^ alone		8.5	0.5 to 16.5
Hypertension^d^ alone		22.3	7.5 to 37.2
Diabetes + Hypertension	Diabetes	6.5	−0.8 to 13.9
	Hypertension	22.0	6.8 to 37.3

(b)			
Indirect effect^a^ through:			
Leptin^b^ alone		59.7	−7.1 to 126.6
Diabetes^c^ alone		9.6	−0.4 to 19.6
Hypertension^d^ alone		22.4	4.2 to 40.6
Diabetes + Hypertension	Diabetes	7.7	−1.3 to 16.7
	Hypertension	22.5	3.6 to 41.4

Results were based on analyses weighted towards the BMI distribution of the general population and were derived from multiplied path coefficients with 95% confidence intervals from general structural equation modeling (path analysis) and expressed as indirect effects in the association between body mass index (kg/m^2^) and chronic kidney disease (defined as having an eGFR < 60 ml/min/1.73m^2^ and/or moderately increased albuminuria) (6a) or expressed as indirect effects in the association between total body fat (%) and chronic kidney disease (6b). Indirect effects were divided by total effects to calculate the percentage mediated.

*CI* confidence interval.

^a^Indirect effects were adjusted for sex, age, smoking, ethnicity, physical activity and Dutch Healthy Diet index

^b^Underwent natural logarithmic transformation

^c^Diabetes mellitus is considered present in case of a self-reported physician’s diagnosis and/or use of glucose lowering drugs or fasting glucose levels ≥ 7 mmol/L

^d^Systolic blood pressure ≥ 140 mmHg and/or the diastolic blood pressure ≥ 90 mmHg or use of antihypertensive drugs

Diabetes and hypertension both also had an indirect effect in the association between BMI or TBF and moderately increased albuminuria. When both diabetes and hypertension were included in the model, the percentage of mediation of the total association between BMI and moderately increased albuminuria was 4.4% (95%CI: 0.2; 8.6) for diabetes and 18.9% (95%CI: 7.3; 30.6) for hypertension (Table 7a). Similarly, the percentage of mediation of the total association between TBF and moderately increased albuminuria was 4.5% (95%CI: −0.1; 9.2) for diabetes and 19.0% (95%CI: 5.5; 32.5) for hypertension (Table 7b). We could not confirm that leptin was a mediating factor in the association of BMI or TBF with moderately increased albuminuria, as it showed no attenuation in the effect in the above analyses (Table 5b).

**Table 7:** (a) Analysis of indirect effects at baseline of the mediators diabetes and hypertension in the association between body mass index and moderately increased albuminuria of 6508 participants of the Netherlands Epidemiology of Obesity (NEO). (b) Analysis of indirect effects at baseline of the mediators diabetes and hypertension in the association between total body fat and moderately increased albuminuria of 6477 participants of the Netherlands Epidemiology of Obesity (NEO)

(a)			
		% of Total Effect	95% CI
Indirect effect^a^ through:			
Diabetes^b^ alone		6.0	1.5 to 10.6
Hypertension^c^ alone		19.2	7.8 to 30.6
Diabetes + Hypertension	Diabetes	4.4	0.2 to 8.6
	Hypertension	18.9	7.3 to 30.6

(b)			
Indirect effect^a^ through:			
Diabetes^b^ alone		6.2	0.8 to 11.5
Hypertension^c^ alone		19.0	5.9 to 32.1
Diabetes + Hypertension	Diabetes	4.5	−0.1 to 9.2
	Hypertension	19.0	5.5 to 32.5

Results were based on analyses weighted towards the BMI distribution of the general population and were derived from multiplied path coefficients with 95% confidence intervals from general structural equation modeling (path analysis) and expressed as indirect effects in the association between body mass index (kg/m^2^) and moderately increased albuminuria (defined as urine albumin-to-creatinine ratio ≥2.5 mg/mmol in men and ≥3.5 mg/mmol in women) (7a) or expressed as indirect effects in the association between total body fat (%) and moderately increased albuminuria (7b). Indirect effects were divided by total effects to calculate the percentage mediated.

*CI* confidence interval

^a^Indirect effects were adjusted for sex, age, smoking, ethnicity, physical activity and Dutch Healthy Diet index

^b^Diabetes mellitus is considered present in case of a self-reported physician’s diagnosis and/or use of glucose lowering drugs or fasting glucose levels ≥ 7 mmol/L

^c^Systolic blood pressure ≥ 140 mmHg and/or the diastolic blood pressure ≥ 90 mmHg or use of antihypertensive drugs

## Discussion

In this Dutch population-based study of 6671 middle-aged participants, we observed that BMI and TBF were associated with an increased odds of CKD of 8% and 5%, respectively. A 2.7-fold increased level of leptin was associated with a 1.6 increased risk of CKD. Hypertension and diabetes, but not leptin, mediated the association of BMI and TBF with CKD. We observed that the association between BMI or TBF and CKD was 8.5% and 9.6% mediated by diabetes and 22.3% and 22.4% by hypertension, respectively.

Obesity and CKD have been known to co-exist [4, 10, 33], which is also shown by our analysis. We observed that BMI and TBF were both associated with an increased risk of CKD and moderately increased albuminuria. Albuminuria is an established risk factor for CKD progression [34, 35]. In addition, TBF might be an important factor in the etiology of albuminuria. Not only the percentage of TBF seems to be important, but especially the distribution of this fat. Individuals with a central pattern of body fat, compared to non-central obesity, are observed to be at greater risk of moderately increased albuminuria [12, 36, 37]. However, in our cross-sectional analysis, there was only a weak positive association between BMI, TBF and eGFR < 60 ml/min/1.73m^2^. As eGFR levels in this cohort of patients are still within the normal range (mean eGFR 86.2 ± 12.4 mL/min/1.73 m^2^), this may be a reason for the weak association we observed.

Diabetes and hypertension are well-known comorbidities of obesity and the most frequent causes of CKD [38, 39]. We observed that higher BMI or TBF were associated with an increased odds of diabetes and hypertension. In addition, having diabetes or hypertension was associated with more than 2-fold increased odds for CKD.

Our findings that BMI and TBF are positively related to leptin concentrations and negatively to adiponectin concentrations, are in line with findings from a previous study where they already confirmed that higher TBF is strongly associated with higher leptin concentrations. In addition, higher visceral adipose tissue was associated with reduced adiponectin concentrations [22]. Adiponectin is an anti-inflammatory protein and has been considered as a marker of kidney injury, but results are conflicting [40]. A study performed on rats with diabetes, showed that overexpression of adiponectin was associated with lower levels of proteinuria and less enlarged kidneys [41]. In contrast, other studies showed a positive association between serum adiponectin levels and mortality in CKD. Unfortunately, the underlying mechanism of this paradox is still unclear [40]. In our study, there was no association between adiponectin and CKD. This can be attributed to the fact that our cohort comprises mainly relatively healthy individuals, with a low prevalence of CKD of 4%. This is in line with a prospective study among elderly Japanese people without CKD, that showed no association between serum adiponectin levels and eGFR decline [42]. In contrast, we did find that higher leptin levels were associated with a small increased risk of CKD. Previous research showed that infusion of leptin into healthy rats for 3 weeks, caused proteinuria due to the development of focal glomerulosclerosis [43]. On the other hand, leptin is mainly cleared by the kidney, therefore serum concentrations are increased in patients with kidney failure and those undergoing dialysis. Taken together, the kidney might be both involved in leptin metabolism, as well as a target for the detrimental effects of leptin in obesity [44, 45].

After adjustment for leptin, hypertension and diabetes separately, we observed the largest attenuation of the association between BMI, TBF and moderately increased albuminuria after adjustment for hypertension and diabetes compared with adjustment for leptin. Diabetes and hypertension are known comorbid conditions of obesity and thought to mediate the effect between obesity and its deleterious renal consequences [10]. We showed that each 1% increase in TBF was associated with a 9% increase in leptin levels, as previously described by Christen, et al. [22]. However, we found no mediation role for leptin between BMI or TBF and CKD or moderately increased albuminuria in our relative healthy cohort. Future research should focus on the interrelation between TBF, serum leptin levels and CKD and its precise mechanism.

The strength of this study is the large number of participants with extensive phenotyping at baseline, as well as data on leptin and adiponectin concentrations. Nevertheless, this study has limitations. Since this study has an observational design, no causal inferences can be made. However, since it is well known that higher TBF is related with higher secretion of leptin [22, 46], combined with our finding that higher TBF increased risk of CKD, it is plausible that higher leptin may cause CKD. In addition, the majority of this study population was Caucasian, therefore the results of this study need to be confirmed in other ethnic groups.

In conclusion, our results suggest that the relations between BMI or TBF with CKD and moderately increased albuminuria are in part mediated by diabetes and hypertension. However, there are signs that kidney damage might be induced by endocrine activity of the adipose tissue via production of leptin. Future studies need to investigate the possible mechanism behind the association between leptin and CKD and the possible role of leptin in the relation between body fat and CKD.

### Supplementary information

Supplementary Information

## Data Availability

As our data could be used to identify individuals, privacy concerns prevent us from allowing them to be publicly available. Nonetheless, data will be available (conditional on agreement on privacy matters and appropriate usage of the data) upon request to the Department of Clinical Epidemiology of the Leiden University Medical Center (Data manager: Ingeborg de Jonge, Data management office, Department of Clinical Epidemiology C7-P, Leiden University Medical Center, P.O. Box 9600, 2300 RC Leiden, The Netherlands, email: i.de_jonge@lumc.nl).
