# Supplementary material for: Hypertension and diabetes, but not leptin and adiponectin, mediate the relationship between body fat and chronic kidney disease
Source: Endocrine. 2024 Apr 16;85(3):1141–53. doi: 10.1007/s12020-024-03811-6 (PMC11316711; doi:10.1007/s12020-024-03811-6)
Supplement: Supplementary file 1 — Supplementary Information [file 12020_2024_3811_MOESM1_ESM.docx]

**Hypertension and diabetes, but not leptin and adiponectin, mediate the relationship between body fat and chronic kidney disease**

Robin Lengton^1,2^, Friedo W. Dekker^1^, Elisabeth F.C. van Rossum^2^, Johan W. de Fijter^3^, Frits R. Rosendaal^1^, Ko Willems van Dijk^4^, Ton J. Rabelink^3^, Saskia Le Cessie^1,5^, Renée de Mutsert^1^ and Ellen K. Hoogeveen^1,3,6^

^1^ Department of Clinical Epidemiology, Leiden University Medical Center, Leiden, The Netherlands

^2^ Department of Internal Medicine, division of Endocrinology, Erasmus University Medical Center Rotterdam, Rotterdam, The Netherlands

^3^ Department of Nephrology, Leiden University Medical Center, Leiden, The Netherlands

^4^ Department of Human Genetics and Medicine, division of Endocrinology, Leiden University Medical Center, Leiden, The Netherlands

^5^ Department of Biomedical Data Sciences, Leiden University Medical Center, Leiden, The Netherlands

^6^ Department of Nephrology, Jeroen Bosch Hospital, Den Bosch, The Netherlands

**Corresponding author:** Robin Lengton, Department of Clinical Epidemiology, Leiden University Medical Center (Building 1, C7-112), Albinusdreef 2, 2333 ZA Leiden, The Netherlands. E-mail: [r.lengton@lumc.nl](mailto:r.lengton@lumc.nl)

**Supplementary table 1.** P-values for interaction at baseline of leptin, diabetes and hypertension with body mass index or total body fat in the association with chronic kidney disease and moderately increased albuminuria of participants of the Netherlands Epidemiology of Obesity (NEO).

|  | | Leptin^a^ (μg/L) | Diabetes^b^ | Hypertension^c^ |
| --- | --- | --- | --- | --- |
|  | | P for interaction | P for interaction | P for interaction |
| *BMI (kg/m^2^)* | |  |  |  |
|  | **Chronic kidney disease^d^** | 0.609 | 0.758 | 0.568 |
|  | |  |  |  |
| *Total body fat (%)* | |  |  |  |
|  | **Chronic kidney disease^d^** | 0.246 | 0.742 | 0.443 |
|  |  |  |  |  |
|  |  |  |  |  |
| *BMI (kg/m^2^)* | |  |  |  |
|  | **Moderately increased albuminuria^e^** | 0.128 | 0.679 | 0.588 |
|  |  |  |  |  |
| *Total body fat (%)* | |  |  |  |
|  | **Moderately increased albuminuria^e^** | 0.861 | 0.865 | 0.285 |

Results were based on analyses weighted towards the BMI distribution of the general population (N=1758), adjusted for sex, age, smoking, ethnicity, physical activity and Dutch Healthy Diet index.
^a^ Underwent natural logarithmic transformation. ^b^ Diabetes mellitus is considered present in case of a self-reported physician’s diagnosis and/or use of glucose lowering drugs or fasting glucose levels ≥7 mmol/L.
^c^ Systolic blood pressure ≥140 mmHg and/or the diastolic blood pressure ≥90 mmHg or use of antihypertensive drugs.
^d^ Defined as having an eGFR <60 mL/min/1.73 m^2^ and/or moderately increased albuminuria.
^e^ Moderately increased albuminuria is defined as urine albumin-to-creatinine ratio ≥ 2.5 mg/mmol in men and ≥ 3.5 mg/mmol in women.
Abbreviations: BMI, body mass index.
